# High-Performance PEDOT:PSS/Hexamethylene Diisocyanate-Functionalized Graphene Oxide Nanocomposites: Preparation and Properties

**DOI:** 10.3390/polym10101169

**Published:** 2018-10-20

**Authors:** José Antonio Luceño Sánchez, Rafael Peña Capilla, Ana Maria Díez-Pascual

**Affiliations:** 1 Department of Analytical Chemistry, Physical Chemistry and Chemical Engineering, Faculty of Biology, Environmental Sciences and Chemistry, Alcalá University, 28871 Madrid, Spain; jose.luceno@uah.es; 2 Department of Signal Theory and Communication, Alcalá University, 28805 Madrid, Spain; rafa.pena@uah.es

**Keywords:** PEDOT:PSS, graphene oxide, nanocomposite, hexamethylene diisocyanate, functionalization degree, sheet resistance, optical transmittance, mechanical properties

## Abstract

Graphene oxide (GO) has emerged as an ideal filler to reinforce polymeric matrices owing to its large specific surface area, transparency, flexibility, and very high mechanical strength. Nonetheless, functionalization is required to improve its solubility in common solvents and expand its practical uses. In this work, hexamethylene diisocyanate (HDI)-functionalized GO (HDI-GO) has been used as filler of a conductive polymer matrix, poly(3,4-ethylenedioxythiophene):poly(styrenesulfonate) (PEDOT:PSS). The nanocomposites have been prepared via a simple solution casting method, and have been characterized by scanning electron microscopy (SEM), UV–Vis and Raman spectroscopies, X-ray diffraction (XRD), thermogravimetric analysis (TGA), tensile tests, and four-point probe measurements to get information about how the HDI-GO functionalization degree (FD) and the HDI-GO concentration in the nanocomposite influence the final properties. SEM analysis showed a very homogenous dispersion of the HDI-GO nanosheets with the highest FD within the matrix, and the Raman spectra revealed the existence of very strong HDI-GO-PEDOT:PSS interactions. A gradual improvement in thermal stability was found with increasing HDI-GO concentration, with only a small loss in transparency. A reduction in the sheet resistance of PEDOT:PSS was found at low HDI-GO contents, whilst increasing moderately at the highest loading tested. The nanocomposites showed a good combination of stiffness, strength, ductility, and toughness. The optimum balance of properties was attained for samples incorporating 2 and 5 wt % HDI-GO with the highest FD. These solution-processed nanocomposites show considerably improved performance compared to conventional PEDOT:PSS nanocomposites filled with raw GO, and are highly suitable for applications in various fields, including flexible electronics, thermoelectric devices, and solar energy applications.

## 1. Introduction

Recently, conductive films comprising conductive polymers [1], carbon-based nanomaterials [2], and hybrid nanocomposites [3] have attracted a lot of interest for applications in several fields, including transparent electrodes, supercapacitors, light-emitting diodes (LEDs), solar cells, thermoelectric devices, and so forth [3,4,5,6]. The main motivation is the reduction in manufacturing costs and the replacement of costly oxides, like indium tin oxide (ITO). In particular, conductive polymers show great potential for the aforementioned applications owing to their exceptional optical and electrical properties combined with their light weight, low cost, flexibility, and outstanding processability [7]. Amongst the conductive polymers, poly(3,4-ethylenedioxythiophene) (PEDOT), composed of ethylenedioxythiophene (EDOT) monomers, is a very promising material owing to its good electrochemical properties, inexpensiveness, and high transparency. However, it is insoluble in numerous common solvents and oxidizes rapidly under air. A polyelectrolyte solution, poly(styrenesulfonate) (PSS), is usually mixed with PEDOT to improve its processability, leading to aqueous PEDOT:PSS dispersions in which conjugated PEDOT polymer carries positive charges and PPS comprises negatively charged sulfonyl groups that serve as counter-ions. Owing to its very high conductivity, processing simplicity, and superior chemical and electrochemical stability, as well as excellent dispersibility in various solvents, PEDOT:PSS has been widely used as conductive material for flexible electronic and optoelectronic devices [8], such as sensors, hole-injection layer of organic LEDs, antistatic coatings, transistors, and flexible electrodes. Nonetheless, PEDOT:PSS presents some disadvantages, like high acidity, hygroscopic character, and inhomogeneous electrical properties, resulting in low durability [2]. 

Graphene oxide (GO), an atomically thick layer of sp^2^ bonded carbon atoms synthesized by the oxidation of graphite, has gained a lot of attention as nanoscale material [9]. This oxidized form of graphene can be processed in aqueous media, it is amphiphilic and biocompatible, and can interact with biological cells and tissues [10]. It is highly hydrophilic since it includes oxygenated functional groups, namely epoxide, hydroxyl, and carbonyl on the basal plane and carboxyl acids on the edges, and can yield stable dispersions in water and other solvents [11], which is essential for a variety of applications. Further, it can be easily functionalized via the surface oxygenated groups, which makes it a highly versatile nanomaterial. The properties of GO significantly differ from those of graphene: The sp^3^ carbon atoms in GO augment the interlayer spacing, thereby enhancing its capability to retain compounds. Besides, the anchored groups and lattice defects act as scattering centers that alter the electronic structure of graphene, and, consequently, modify the electrical transport. Thus, GO exhibits lower electron mobility than graphene, with a sheet resistance of about 10^12^ Ω/sq or higher [12]. GO can be synthesized by four main routes [9,13]: Staudenmaier, Hofmann, Brodie, and Hummers. Numerous variations of these approaches have been reported to attain better results and cheaper processes [14]. This carbon-based nanomaterial presents excellent strength and unique optical and electronic properties combined with high flexibility, transparency, and excellent stability [15,16,17].

Several strategies have been reported to prepare GO/polymer nanocomposites, including physical and chemical approaches [16,17]. The physical (or non-covalent) methods are generally preferred, since they do not perturb the π-π conjugated system of the GO hexagonal network, and therefore do not affect the final properties of the composite [18]. These include solution mixing, melt-blending, and in situ polymerization. The solution technique involves the dispersion of both the carbon-based nanomaterial and the polymer in an appropriate solvent. On the other hand, the melt-blending process is not commonly used, since it requires the blending of GO into a molten polymer matrix under intense shearing. Nanocomposites with conductive polymers can also be fabricated by in situ polymerization [19], in which GO is firstly swollen by the monomer, and upon addition of the initiator, the polymerization starts by either heat or radiation. The chemical methods involve the covalent bonding (grafting) of polymer chains onto GO, and can be performed by means of “grafting to” or “grafting from” routes [17]. In the former, a polymer with a reactive functional groups is attached to the GO surface via addition reactions; its major drawback is that the percentage of grafted polymer is typically low owing to its low reactivity and high steric hindrance. Conversely, in the “grafting from” strategy, the polymer chains are grown from the GO surface via in situ polymerization of the monomers [20]; this procedure is more effective and tailorable, leading to nanocomposites with a higher percentage of grafting.

Over the last years, several studies dealing with graphene (or its derivatives)/PEDOT:PSS nanocomposites have been published [21,22,23,24]. The composites can be fabricated via four main strategies: (a) The simple mixing of GO and PEDOT:PSS [21], which allows GO contents higher than 10%. However, the homogeneous dispersion of graphene in PEDOT:PSS is restrained since the nanomaterial is easily bent and PEDOT:PSS nanoparticles (30–50 nm size) are in colloidal state; (b) mixing of GO and PPS followed by in situ polymerization of the EDOT monomer [22]. Nonetheless, this strategy includes a reduction process before polymerization and the resulting product must be redispersed in solvent, leading to a low conductivity. Further, the reducing agents (i.e., hydrazine) are highly toxic; (c) use of a dispersing agent for the exfoliation of GO, and mixing of the dispersed graphene with PEDOT:PSS [23]. The main disadvantage of this approach is that the dispersing agents can cause gelation of the solution, thereby worsening the solution stability; and (d) direct mixing of reduced graphene oxide (rGO) with PEDOT:PSS [24], albeit it is difficult to homogeneously disperse rGO sheets in the polymer due to their low solubility in aqueous solution. Therefore, the development of a novel inexpensive, simple, environmentally friendly, and large-scale process for the fabrication of highly conductive PEDOT:PSS/graphene nanocomposites is highly desirable.

In a previous study [25], hexamethylene diisocyanate (HDI)-functionalized GO was synthesized via a modified Hummers’ method followed by reaction with HDI in the presence of triethylamine (TEA) as a catalyst. Several reactions conditions were tried to enhance the functionalization degree. The resulting HDI-GO nanomaterials were more hydrophobic than raw GO and were easily suspended in polar aprotic solvents, like dimethylformamide (DMF), N-methylpyrrolidone (NMP), and dimethyl sulfoxide (DMSO). Herein, we report for the first time that improved dispersion of GO nanosheets into PEDOT:PSS can be attained via previous functionalization of the carbon nanomaterial with organic HDI. Enhancing the dispersion of GO within the conducting polymer matrix is critical to achieve improved electrical, thermal, optical, and mechanical properties. The PEDOT:PSS/HDI-functionalized GO nanocomposites have been prepared via a simple and easily scalable solution casting method, and have been characterized by a number of techniques to get insight about how the HDI-GO functionalization degree (FD) and the HDI-GO weight percentage (wt %) in the nanocomposite influence the final properties. The optimum balance of properties was attained for samples with 2 and 5 wt % HDI-GO (FD = 18.1%). These solution-processed nanocomposites are highly suitable for applications in various fields, including conventional panel displays, solar cells, touch panels, and so forth.

## 2. Materials and Methods

### 2.1. Reagents

Orgacon ™ DRY, grey-blue re-dispersible pellets of poly(3,4-ethylenedioxythiophene) poly(styrenesulfonate) (PEDOT:PSS ratio of 1:2.5, d_25°C_ = 1.911 g/cm^3^), triethylamine (TEA, >98%, *M*_w_ = 101.193 g/mol), H_2_SO_4_, KMnO_4_, P_2_O_5_, K_2_S_2_O_8_, and H_2_O_2_ (30 wt % in water) were obtained from Sigma-Aldrich (Madrid, Spain) and used as received. Graphite powder was purchased from Bay Carbon, Inc. (Bay City, MI, USA). Hexamethylene diisocianate (HDI, >99%, *M*_w_ = 168.196 g/mol) was provided by Acros Organics (Madrid, Spain). All the organic solvents were HPLC grade and were purchased from Scharlau S.L. (Barcelona, Spain).

### 2.2. Synthesis of Hexamethylene Diisocyanate-Functionalized Graphene Oxide (HDI-GO)

HDI-GO was prepared according to the procedure described in our previous work [25]. Briefly, GO was first synthesized using a modified Hummers’ method from flake graphite [11], by heating graphite powder, H_2_SO_4_, K_2_S_2_O_8_, and P_2_O_5_ at 80 °C for 5 h, followed by oxidation for a second time via addition of H_2_SO_4_, KMnO_4_, and water in an ice-water bath. After several cycles of centrifugation and purification, it was vacuum freeze-dried before use. 

The functionalization process was performed by weighing the desired amount of GO powder and placing it into a round-bottom flask, followed by addition of dried toluene under Ar atmosphere. The dispersion was ultrasonicated for 2 h, and then a TEA catalyst and HDI were added dropwise (GO:HDI:TEA weight ratio of 1:1:1). The mixture was heated to 60 °C and stirred at 350 rpm for 12 h under an inert atmosphere. The resulting product (HDI-GO 1) was precipitated by pouring it into methylene chloride, filtered, purified by washing thoroughly with methylene chloride, and dried under vacuum; it showed a functionalization degree (FD) of 12.3% as determined by elemental analysis [25]. In another synthesis, to improve the GO exfoliation, the bath sonication was combined with 3 probe sonication cycles (5 min on/5 min off, 40% amplitude), leading to a product (HDI-GO 6) with an FD close to 18.1%. 

### 2.3. Synthesis of PEDOT:PSS/HDI-Functionalized GO Nanocomposites

**Scheme 1:**
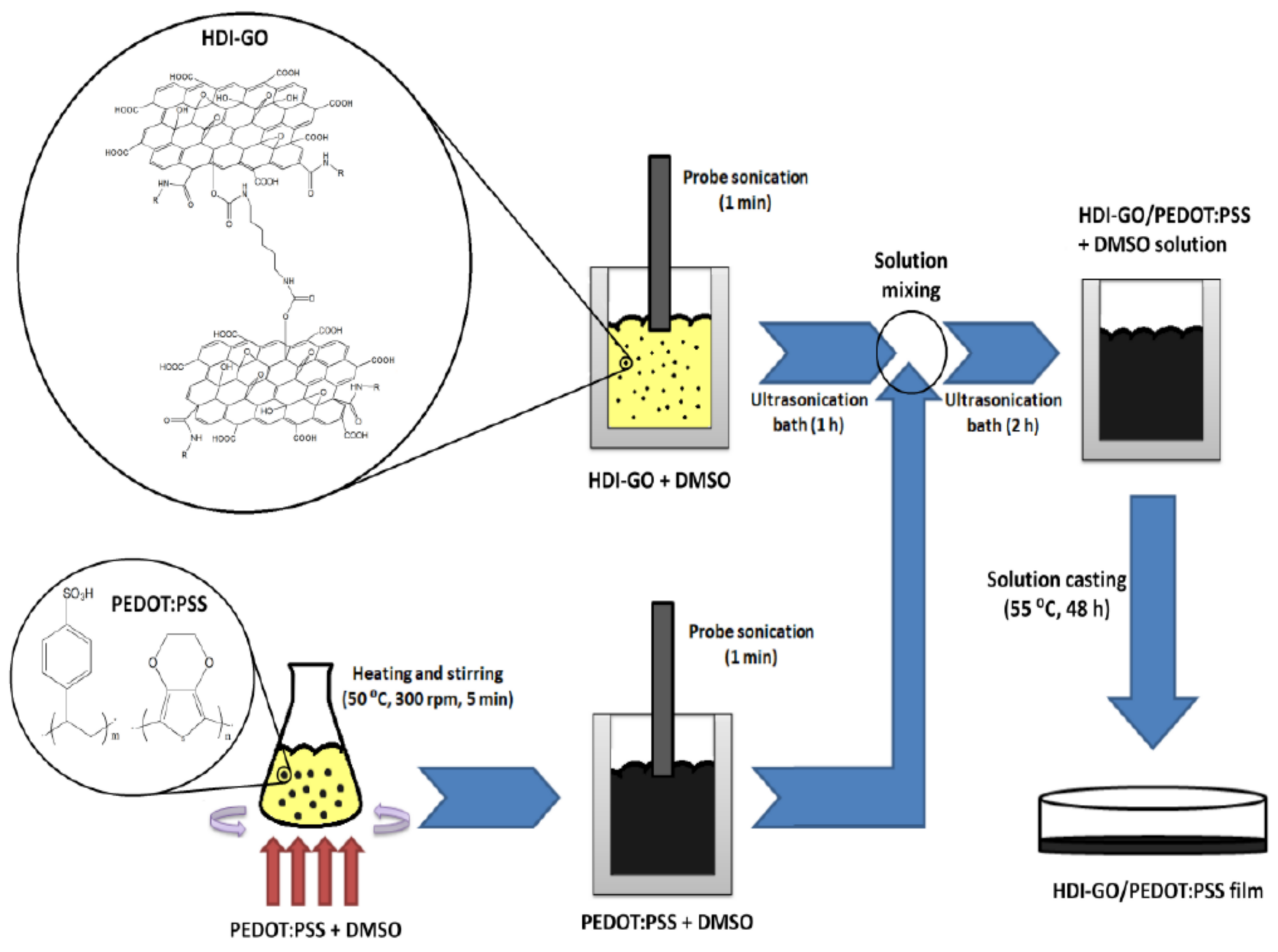
Schematic representation of the synthesis procedure of PEDOT:PSS/HDI-functionalized GO nanocomposites.

The nanocomposites were prepared via the solution casting method. Firstly, the desired amount (0.25, 0.5, 1.0, 2.5, and 5.0 mg) of HDI-GO was dispersed in 2.5 mL of DMSO by probe sonication for 1 min at a 40% amplitude followed by ultrasonication in an ultrasonic bath for 1 h to create a homogeneous suspension of HDI-GO nanosheets in the solvent. Separately, the polymer (0.05 mg for each sample) was dissolved in 5 mL of DMSO by heating at 50 °C and stirring at 300 rpm for 5 min combined with probe sonication for 1 min. Then, the PEDOT:PSS solution was added to the HDI-GO suspensions to obtain different HDI-GO/PEDOT:PSS weight ratios (0.5, 1.0, 2.0, 5.0, and 10 wt %), and the mixture was bath sonicated for another 2 h, leading to homogenous dispersions, without precipitates (Figure S1 in the Supplementary Material). Afterward, the samples were poured onto Petri dishes and then were dried off in a furnace at 55 °C for 48 h to eliminate the residual solvent. Finally, the films were removed from the Petri dish using a little alcohol, and were dried again under vacuum (≈5 Torr) at 60 °C for 5 h to ensure the complete removal of DMSO. An average film thickness of 13 ± 2 μm was measured using a surface profiler. A schematic representation of the synthesis procedure of the nanocomposites is shown in Scheme 1.

For comparative purposes, reference samples containing neat PEDOT:PSS (0 wt % HDI-GO) and 5.0 mg of raw GO (10 wt % GO) were prepared in a similar way. Further, to investigate the influence of the level of functionalization of HDI-GO on the nanocomposite properties, two sets of composites were prepared, one using HDI-GO 1 (FD = 12.3%) and the other with HDI-GO 6 (FD = 18.1%). To simplify the nomenclature, the nanocomposites will be designated as P/GO or P/HDI-GO, with their nanofiller content in brackets. Homogeneous films were obtained for all the HDI-GO concentrations and both levels of functionalization studied, as shown in Figure S2 in the Supplementary Material. With increasing HDI-GO content, the transparency of the films decreased, as the colour changed from metallic grey to black. 

### 2.4. Intrumentation

Samples were weighed using a Sartorious Cubis^®^ Ultramicro Balance (Leicestershire, UK) with readability of 0.0001 mg. A Selecta 3001208 ultrasonic bath (Madrid, Spain) and a 24 kHz Hielscher UP400S ultrasonic tip (Teltow, Germany) with a maximum power output of 400 W, equipped with a titanium sonotrode (Ø = 7 mm, l = 100 mm) were used to prepare the PEDOT:PSS and HDI-GO dispersions in DMSO. 

The morphology of the nanocomposites was analyzed with an SU8000 Hitachi scanning electron microscope (SEM, Hitachi, Ltd., Tokyo, Japan). The accelerating voltage was set at 15.0 kV and the emission current was 10 mA. Prior to the observations, the films were cryofractured in liquid nitrogen and then coated with a ~5 nm Au:Pd overlayer to prevent charge accumulation during electron irradiation.

Room temperature Raman spectra were recorded with a Renishaw Raman microscope (Gloucestershire, UK) equipped with a He-Ne laser (632.8 nm), at a laser power of 1 mW. Thirty scans were obtained for each sample to reduce the signal-to-noise ratio. The Raman spectra were processed using the WiRE 3.3 Renishaw software. For comparative purposes, spectra were normalized to the G band.

The thermal stability of the nanocomposites under nitrogen atmosphere was analyzed by thermogravimetric analysis (TGA) using a TA Instruments Q50 thermobalance (Barcelona, Spain) at a heating rate of 10 °C/min, from room temperature to 700 °C, under a purge gas flow rate of 60 mL/min. Samples of ~5 mg were placed into alumina pan for the tests after drying for 72 h.

X-ray diffraction (XRD) analysis was carried out with a Bruker D8 Advance diffractometer (Karlsruhe, Germany) using a Cu tube as the X-ray source (λ CuKα = 1.54 Å), with a voltage of 40 kV and intensity of 40 mA.

The optical transmittance of the films was measured at room temperature with a UV–Vis spectrophotometry (JASCO Corporation, Tokyo, Japan, model V-650), in the wavelength range of 250–800 nm.

Tensile testing was carried out with an Instron 5565 Testing Machine (Norwood, MA, USA), using a 1 kN load cell and at a crosshead speed of 10 mm/min, under ambient conditions. The results reported are the mean values for six replicates. 

The sheet resistance of the nanocomposites was measured using a four-point probe resistivity measurement system (Multiheight Probe station, Leighton Buzzard, UK) with an automatic Z motion. The voltage and the electrical current were measured using a KEITHLEY 2182A nanovoltmeter (Bracknell, UK) and a KEITHLEY 6221 current source, respectively. The sheet resistance (Rs) was determined as: Rs = 4.532 × (*V*/*I*), where *V* is the test voltage and *I* is the current. At least 5 measurements at different points of each nanocomposite film were performed to ensure reproducibility, and the average values are reported.

## 3. Results and Discussion

### 3.1. Morphology of PEDOT:PSS/HDI-GO Nanocomposites

**Figure 1:**
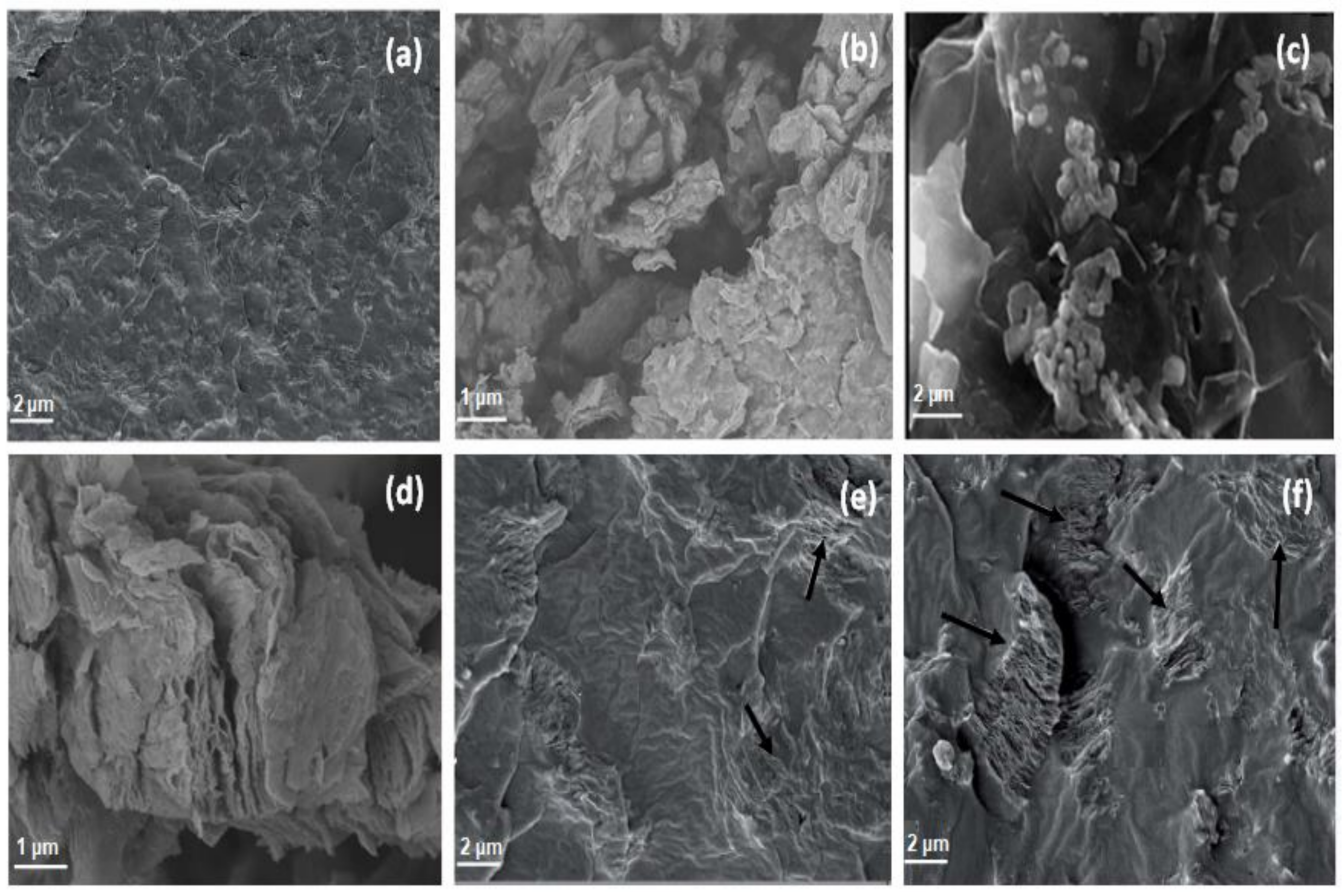
Representative SEM micrographs of neat PEDOT:PSS (**a**), raw GO (**b**), P/GO (10 wt %) (**c**), HDI-GO 6 (**d**), P/HDI-GO 6 (0.5 wt %) (**e**), and P/HDI-GO 6 (10 wt %) (**f**). The arrows show HDI-GO nanosheets embedded within the polymer matrix.

The surface morphology of the neat polymer, GO, HDI-GO, and the nanocomposites was assessed by SEM, and some representative micrographs are compared in Figure 1. The neat polymer (Figure 1a) shows a homogenous slightly rough surface, with good film-forming capability. Raw GO appears quite compact and aggregated (Figure 1b), and is composed of wrinkled and highly flexible graphene flakes, with thicknesses in the range of 10–50 nm and lateral sizes of several micrometers. Conversely, HDI-GO 6 (Figure 1d) appears as stacked graphene nanosheets with a smoother surface, attributed to the grafting of the HDI chains on the GO surface. The sheets are less bendable, probably due to the wrapping effect of the alkyl organic chains that cover the wrinkles, and appear thicker, with thicknesses of up to 80 nm. Similar morphology was found for HDI-GO 1, albeit with thinner sheets, corroborating that the flake thickness increases with increasing FD.

**Scheme 2:**
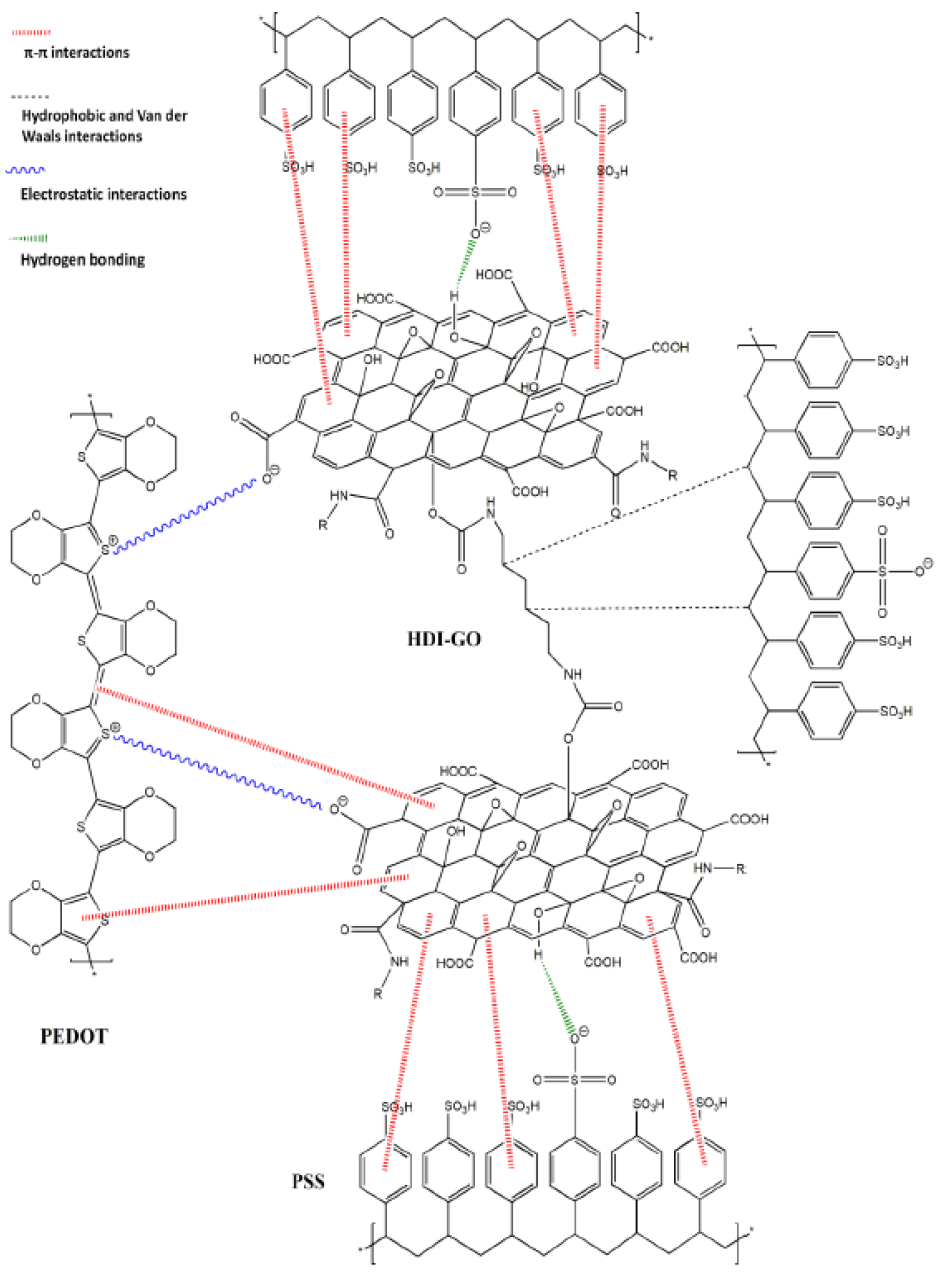
Representation of the different types of interactions between PEDOT:PSS and HDI-GO.

Regarding the nanocomposites, a high degree of agglomeration of the graphene layers can be observed in P/GO (10.0 wt %) due to intense van der Waals forces between the nanosheets (Figure 1c). It was not possible to homogeneously disperse the hydrophilic GO sheets in the polymer due to their poor dispersibility in DMSO, the polar aprotic solvent used for the solution casting process. Thus, numerous graphene bundles heterogeneously distributed throughout the PEDOT:PSS matrix can be found, which is an obstacle to the formation of homogenous conductive paths, and hence to improvement of the electrical conductivity. Further, the nanocomposite surface is quite smooth and plain. In contrast, P/HDI-GO 6 (10 wt %) displays randomly dispersed graphene sheets. (Figure 1f, see the arrows on the micrograph). The reduction in the strength of hydrogen bonding between the GO nanosheets upon functionalization with HDI makes the HDI-GO surface more hydrophobic than that of raw GO [25], hence it was well dispersed in DMSO by the combination of probe and bath sonication used herein. Consequently, PEDOT:PSS was able to diffuse within the stacked and well exfoliated structure of HDI-GO, leading to the formation of a thin polymer coating on the graphene layers. The π-π interactions between HDI-GO and PEDOT:PSS (Scheme 2) promote the formation of a tightly coated polymeric layer on the graphene surface. Further, the sample surface is coarser compared to that of neat PEDOT:PSS, attributed to the polymer matrix distortion in the presence of the HDI-GO reinforcement. Similar morphology, albeit with fewer and more individual HDI-GO sheets, was observed for P/HDI-GO 6 (0.5 wt %), Figure 1e, as well as for the rest of the HDI-GO 6 nanocomposites. 

Analogous surface morphology was also found for the nanocomposites incorporating HDI-GO 1 (Figure S3 in the Supplementary Material), although the graphene sheets showed a more bent appearance, slightly lower level of exfoliation, and poorer impregnation by the polymer compared to samples reinforced with HDI-GO 6. This indicates that the higher the HDI-GO functionalization degree, the more hydrophobic its character, hence the better its dispersibility in DMSO, and, consequently, the more homogenous its dispersion within the PEDOT:PSS matrix.

It is worthy to note that the variations in surface morphology amongst the diverse nanocomposites are likely related to the different interactions between PEDOT:PSS and the different graphene derivatives. The PEDOT chains have numerous π bonds, as well as the aromatic rings of GO and HDI-GO, hence both graphene-based nanomaterials can interact with the matrix by π-π stacking as well as via electrostatic interactions between their negatively charged carboxylic acid groups and the positively charged PEDOT chains. Besides, their surface OH groups are prone to interact with the negatively charged sulfonyl groups of PSS via hydrogen bonding. On the other hand, HDI-GO can interact with the alkyl side chains of PSS via hydrophobic interactions and van der Waals forces [26] (see Scheme 2). Thus, HDI-GO can have strong interactions with both PEDOT and PSS chains, and can be better embedded within the matrix, leading to a rougher structure. The higher the FD of HDI-GO, the more intense would be the interactions with the PPS chains, and, consequently, the coarser the surface.

### 3.2. Raman Spectra of PEDOT:PSS/HDI-GO

**Figure 2:**
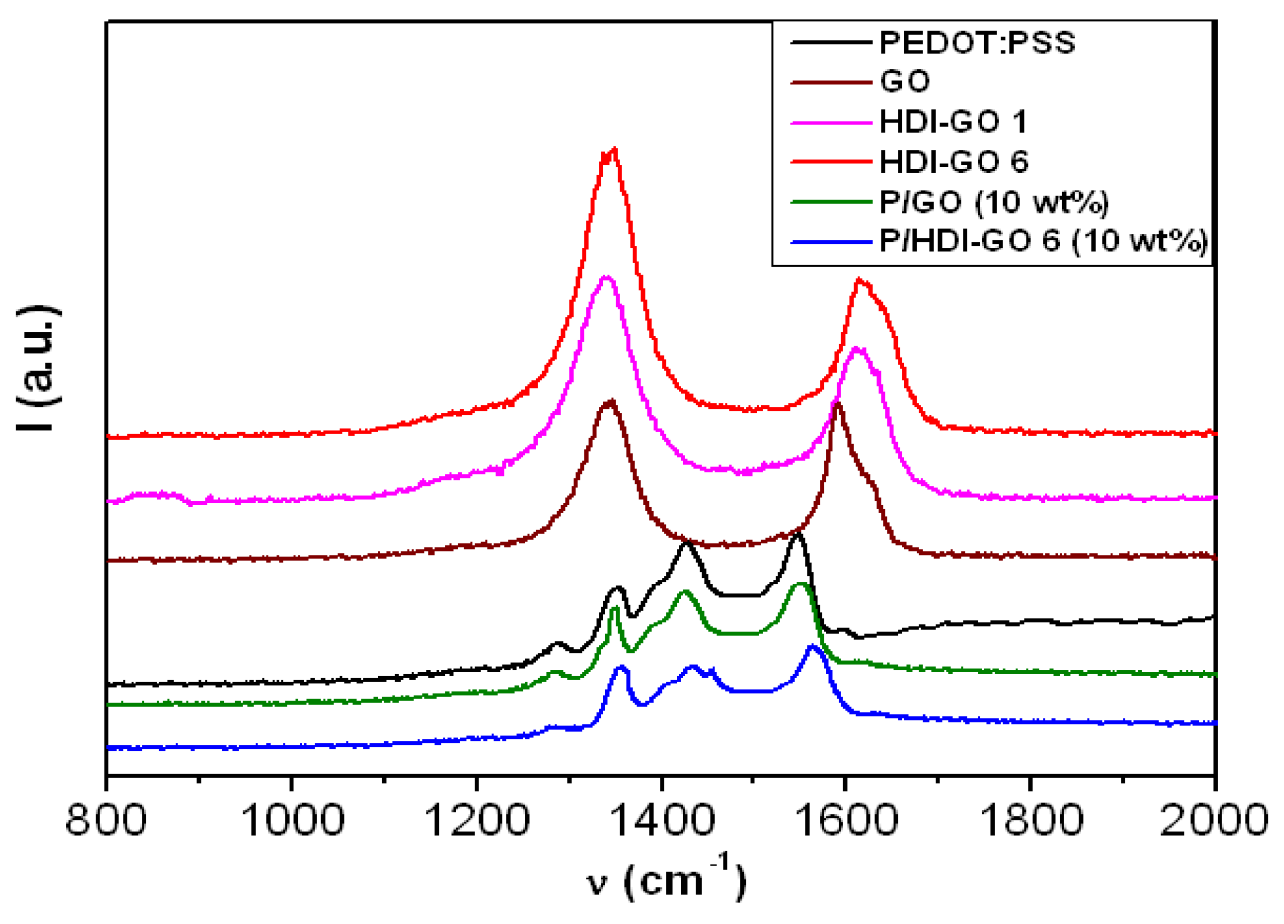
Raman spectra of neat PEDOT:PSS, raw GO, HDI-GO 1, HDI-GO 6, P/GO (10 wt %), and P/HDI-GO 6 (10 wt %).

The chemical structures of PEDOT:PSS, GO, HDI-GO, and the nanocomposites with 10 wt % HDI-GO or GO loading were characterized by Raman spectroscopy as shown in Figure 2. Data obtained from the Raman spectra of the rest of the nanocomposites are tabulated in Table S1 (see the Supplementary Material). 

The spectrum of the neat polymer shows four characteristic stretching vibrations [27]: The C–C inter-ring stretching at 1280 cm^−1^, the single C–C stretching at 1356 cm^−1^, the C=C symmetrical stretching at 1427 cm^−1^, and the C=C antisymmetric stretching at 1550 cm^−1^. On the other hand, the most important features in the spectrum of GO are the disorder induced D band at 1345 cm^−1^ that shows the level of structural disorder, and the tangencial G band at ~1595 cm^−1^ arising from in-plane displacements in the graphene sheets [28]. Similar spectrum is observed for the HDI-GO samples, albeit they show a broadening and shift of the G-band towards higher wavenumbers, ascribed to a change in the electronic structure of GO in the presence of electron-acceptor groups [25]. Further, this upshift can also be related to an increase in defect density, since the position of the G band depends on the concentration of defects in the graphene sheet [29]. The D to G band intensity ratio (*I*_D_/*I*_G_) provides quantitative information about the amount of defects in graphene sheets: The higher the ratio, the greater the disorder [29]. This ratio increases by about 1.55 and 1.73 for HDI-GO 1 and HDI-GO 6, respectively, compared to pristine GO, which evidences a diminution in the structural order upon grafting the HDI chains onto the GO surface. 

Regarding the nanocomposites, a diminution in the intensity of the bands arising from PEDOT:PSS is observed, together with a shift in the position of the peaks towards higher wavenumbers. This phenomena are ascribed to the adsorption of the polymeric chains onto the GO or HDI-GO surface via π-π stacking interactions, leading to the formation of a tightly coated PEDOT:PSS layer on the carbon nanomaterial surface [27]. Focusing on the C=C stretching modes, it is found that the bands in P/GO (10 wt %) are relatively close to those of pristine PEDOT:PSS (Table S1). These small shifts corroborate that the interactions between the polymer chains and GO are not very strong. In contrast, P/HDI-GO 1 (10 wt %) and P/HDI-GO 6 (10 wt %) display significant upshifts in the position of the bands by up to 13 and 17 cm^−1^ in the C=C antisymmetric stretching, respectively. The noticeable upshift of the Raman bands when HDI-GO is mixed with PEDOT:PSS confirms again the strong interactions between the nanocomposite components. Analogous behaviour of the shift of the Raman bands has been reported for PEDOT:PSS hybridized with graphene sheets and multi-walled carbon nanotubes (CNTs) through in situ polymerization [30]. Further, the higher the FD of HDI-GO, the more intense would be the interactions with the PPS chains, and the stronger the upshift of the Raman bands. 

### 3.3. XRD Analysis of the Nanocomposites

**Figure 3:**
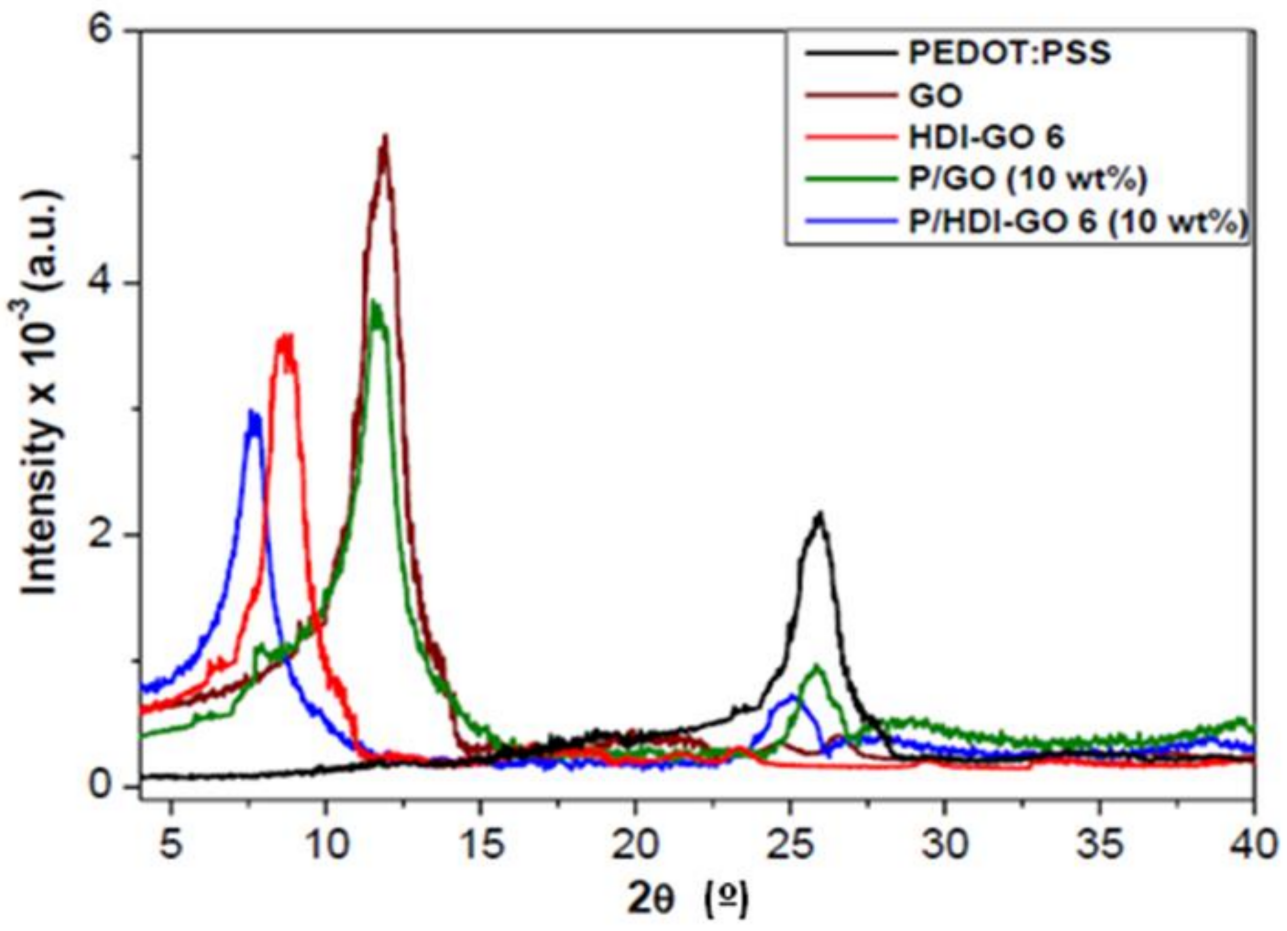
XRD patterns of neat PEDOT:PSS, raw GO, HDI-GO 6, and the corresponding nanocomposites with 10 wt % nanofiller content.

The raw materials and the nanocomposites were also investigated by XRD measurements, and typical patterns of PEDOT:PSS, GO, HDI-GO 6, P/GO (10 wt %), and P/HDI-GO 6 (10 wt %) are compared in Figure 3. Similar diffractograms were obtained for the rest of HDI-reinforced nanocomposites, and the data derived from the diffraction patters are summarized in Table S1 (Supplementary Material). 

Neat PEDOT:PSS has a semicrystalline nature, and shows a peak at 2θ ≈ 25.9°, which arises from the (020) plane of the stacked structure of the polymeric chains, indicating the short domain order of aligned PEDOT chains [31]. This peak can also be observed in the patterns of the nanocomposites, albeit with decreased intensity and shifted towards lower angles. Analogous behaviour of diminution in the peak intensity has been reported for graphene quantum dots/PEDOT:PSS composites [32]. Interestingly, this peak is weaker and appears at a lower 2θ value for the nanocomposites reinforced with HDI-GO compared to that with the same content of GO (see Table S1), which is likely related to the different interaction between the graphene derivatives and the polymer matrix. Thus, the hydrophobicity and strong interaction between HDI-GO and the matrix chains destroys the crystalline region and the ordered alignment of PEDOT:PSS. This is also related to the more uniform dispersion of the HDI-GO, as revealed by SEM analysis, that causes stronger interaction with the polymer chains. Further, for the same HDI-GO content, this (020) peak is less intense and is located at lower angles for the nanocomposites comprising HDI-GO 6 compared to those with HDI-GO 1, which has lower FD and is less hydrophobic. In the nanocomposite with GO, this characteristic peak of PEDOT:PSS is less affected, indicative of weaker interactions with the matrix.

On the other hand, raw GO presents a characteristic peak at 2θ = 11.8° related to the (002) diffraction of the GO sheet [33], from which the interlayer *d* spacing value was calculated to be 0.748 nm according to the Bragg’s equation [34]. For HDI-GO 1 and HDI-GO 6, the reflection is less intense and appears at 2θ = 9.2 and 8.8°, which correspond to an interlayer distance of 0.961 and 1.003 nm, respectively. The rise in the *d* spacing is attributed to the presence of HDI chains intercalated between the GO layers, as has been previously reported for composites with polymeric chains inserted between the GO nanosheets [11,35]. Regarding the nanocomposites, a shift of this (002) peak towards lower 2θ values is also observed compared to either GO or HDI-GO, indicative of an additional enlargement in the interlayer spacing of the carbon nanomaterial. For P/GO (10 wt %), the downshift is fairly small, resulting in a *d* spacing of 0.776, while for P/HDI-GO 1 (10 wt %) and P/HDI-GO 6 (10 wt %), it is more intense, leading to *d* values of 1.108 and 1.165 nm, respectively (Table S1). These noticeable increases of the interlayer spacing in PEDOT:PSS/HDI-GO nanocomposites are attributed to the strong interaction of the polymer chains with the GO nanosheets and their intercalation in between the nanomaterial layers. Again, the higher the FD of HDI-GO, the larger the interlayer distance in the nanocomposites. Overall, the XRD patterns corroborate the effectiveness of the solution casting process developed in this work to separate the layered and stacked GO structure and enable the penetration of the PEDOT:PSS chains within the interlayer spacing.

### 3.4. Thermal Stability of the Nanocomposites

**Figure 4:**
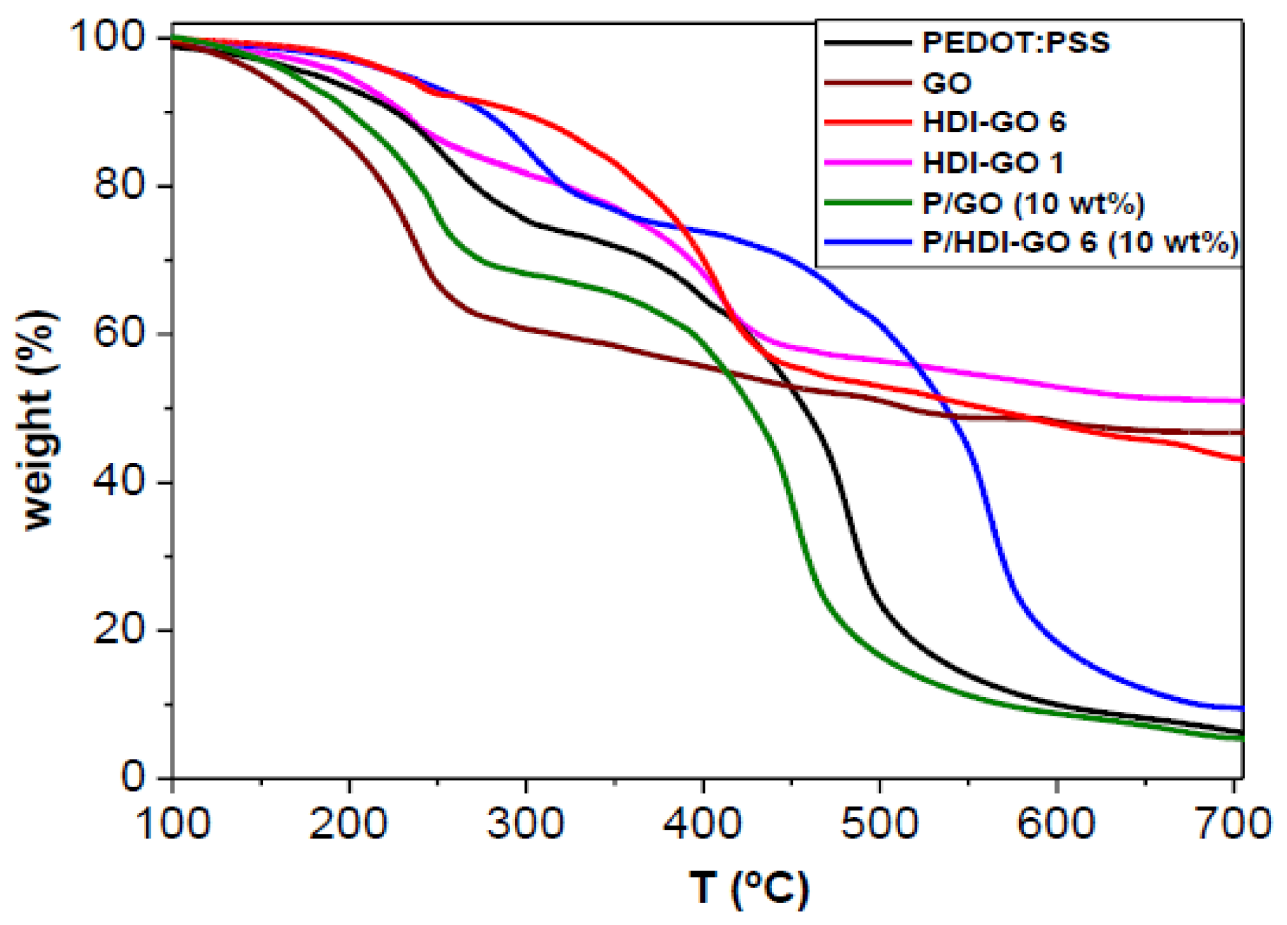
TGA curves under inert atmosphere of neat PEDOT:PSS, raw GO, HDI-GO 1, HDI-GO 6, and the nanocomposites with 10 wt % nanofiller content.

The thermal stability of the PEDOT:PSS/HDI-GO nanocomposite was examined by TGA. Figure 4 shows the weight loss of the raw materials and the nanocomposites with 10 wt % nanofiller content upon heating under a nitrogen atmosphere. The results derived from the rest of the nanocomposites are collected in Table S2 (Supplementary Material).

Neat PEDOT:PSS presents two decomposition stages; the first weight loss up to 300 °C can be ascribed to the decomposition of PSS via rupture of the sulfonate group from styrene [36], and the second loss up to 550 °C is attributed to the rupture of the skeletal PEDOT and/or PSS backbone chain structure [37]. On the other hand, pristine GO displays a one-step degradation process, with a major weight loss below 250 °C ascribed to the decomposition of the surface epoxide, hydroxyl, and carboxylic acid functional groups [25]. Moreover, a small weight loss is found above 250 °C, ascribed to the removal of additional functional groups. Conversely, the HDI-GO nanomaterials present two decomposition stages, the first due to the elimination of the residual oxygenated surface groups, and the second to the decomposition of the HDI chains anchored to the GO surface. The thermal stability of HDI-GO increases with increasing FD (Table S2), attributed to a higher degree of crosslinking between the nanosheets. 

Regarding the nanocomposites, a two-step degradation process is also observed, similar to that of the parent polymer. By adding 10 wt % GO, the onset point hardly increases, and the curve shifts to the lower temperature side, showing that GO negatively influences the thermal stability of PEDOT:PSS. Nonetheless, upon addition of increasing HDI-GO contents, the PEDOT:PSS curve moves gradually towards higher temperatures, the onset point is delayed, and the weight residue is increased (Table S2), indicative of higher thermal stability. In particular, the initial degradation temperature (*T*_i_) at 2% weight loss increased from 130 for the neat polymer to 184 °C for P/HDI-GO 6 (10 wt %), and the temperature of the maximum rate of weight loss (*T*_max_) of the first and the second degradation stages increased by about 53 and 81 °C, respectively. These outstanding enhancements are ascribed to the very homogenous dispersion of the HDI-GO 6 within the PEDOT:PSS matrix, as revealed by SEM images (Figure 1d,e). The crosslinked GO nanosheets randomly and well distributed inside the conductive polymer can act as a barrier and a thermal shielding material to insulate the PEDOT:PSS chains from the heat and hinder the diffusion of the degradation products from the bulk of the composite to the gas phase through the formation of a tortuous path. Analogous behaviour of thermal stability improvement has been reported for polypropylene fumarate (PPF)/polyethylene glycol (PEG)-modified GO [11] and poly(ethylene) (PE)/functionalized GO nanocomposites [38], ascribed to the free radical transfer between the matrix and graphene nanosheets and the barrier effect of functionalized GO. Further, the strong PEDOT:PSS-HDI-GO interactions could constrain the rotational movement of the polymeric chains, thereby reducing the amplitude of the molecules moving under the temperature influence, thus leading to better thermal stability. The comparison of the degradation temperatures of nanocomposites reinforced with HDI-GO 1 and HDI-GO 6 (Table S2) reveals that the thermal stability improves with increasing the FD of HDI-GO (i.e., by an average of 15 °C for the same nanofiller loading), likely due to the higher level of crosslinking between the GO layers and their better impregnation by the polymer, and, as suggested by SEM, that results in a more effective barrier effect. On the other hand, composites with 5 and 10 wt % HDI-GO show approximately the same *T*_i_ and *T*_max_ values, suggesting that the barrier effect imposed by the nanomaterial layers has leveled off, hence further increase in the nanofiller loading would not result in an additional property improvement. TGA results demonstrate that the addition of HDI-GO with a high FD significantly improves the thermal stability of PEDOT:PSS, which is an important result from a practical viewpoint particular for the purpose of photovoltaic application.

### 3.5. Optical Transmittance of the Nanocomposites

**Figure 5:**
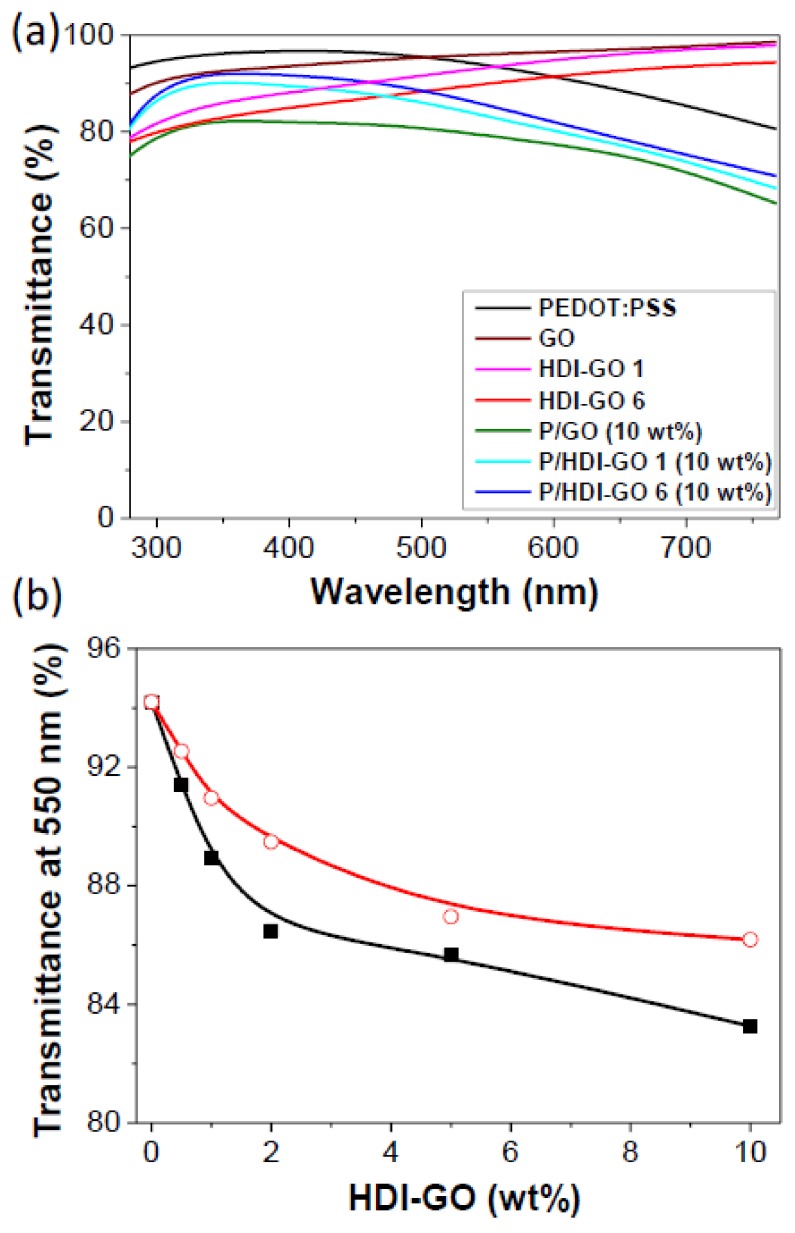
(**a**) Optical transmittance of neat PEDOT:PSS, GO, HDI-GO, and the nanocomposites with 10 wt % loading. (**b**) Optical transmittance at 550 nm of nanocomposites reinforced with HDI-GO 6 (**open circles**) and HDI-GO 1 (**solid squares**) as a function of the HDI-GO content.

The transmittance of neat PEDOT:PSS, raw GO, HDI-GO 1, HDI-GO 6, and the nanocomposites with the highest nanomaterial loading in the wavelength range of 250–800 nm is plotted in Figure 5a. The neat polymer shows a transmittance close to 94% at 550 nm, which decreases smoothly with an increasing wavelength. On the other hand, the transmittance of raw GO at 550 nm is about 96.5%, in agreement with the results reported previously [3], and drops slightly by about 3.5 and 7.2% for HDI-GO 1 and HDI-GO 6, respectively, since the energy position of the density of states can be altered upon the functionalization treatment with organic HDI, thus modifying the optical properties of graphene sheets [39]. Regarding the nanocomposites filled with 10 wt % HDI-GO 1 and HDI-GO 6, a reduction in optical transmittance is found compared to PEDOT:PSS by about 11 and 8% at 550 nm, respectively; however, taking into account the instrumental resolution, the differences in the transmittances are relatively small. A reduction in optical transmittance has also been previously reported for PEDOT:PSS films incorporating CNTs and graphene [40,41]. Nonetheless, upon addition of 10 wt % GO, the reduction in the transmittance of PEDOT:PSS is more significant (i.e., by about 16% at 550 nm, Figure 5a), likely due to the high degree of agglomeration of the graphene layers in the conducting matrix, as revealed by SEM (Figure 1c).

The optical transmittance at 550 nm of PEDOT:PSS reinforced with HDI-GO 1 and HDI-GO 6 as a function of the nanofiller content is plotted in Figure 5b. For both types of nanocomposites, the rise in the HDI-GO concentration steadily reduces the transmittance, with the drop being more pronounced up to 2.0 wt % loading. This behaviour is consistent with the expectation that an increased amount of HDI-GO nanosheets within the PEDOT:PSS would absorb more light. Nonetheless, it is worthy to note that the optical transmittance found herein for the nanocomposites with the highest loading (about 83 and 86% for HDI-GO 1 and HDI-GO 6, respectively) is somewhat higher than that reported for PEDOT:PSS films reinforced with comparable or even higher amounts of CNTs, G, or rGO that is close to 80% [40,41], probably due to the very homogenous nanomaterial dispersion and the strong PEDOT:PSS-HDI-GO interfacial adhesion attained via the HDI functionalization treatment followed by the solution casting processing developed in this work. More importantly, the values obtained for nanocomposites with HDI-GO 6 loadings in the range of 2–10 wt % are comparable to those reported for indium tin oxide (ITO) thin films, a transparent conductive electrode widely used in solar cells, coated on a glass substrate (90%) or on flexible plastic substrates (85%) [42]. Interestingly, the transmittance of composites filled with HDI-GO 6 is higher than that of composites with the same amount of HDI-GO 1, despite the transmittance of HDI-GO decreasing with increasing FD, likely due to the more homogenous dispersion of the GO nanosheets and the stronger interactions with the PEDOT:PSS matrix. From these results, it is inferred that the FD of HDI-GO and its weight percentage in the nanocomposites are important parameters that control the optical transmittance. 

### 3.6. Sheet Resistance of the Nanocomposites

**Figure 6:**
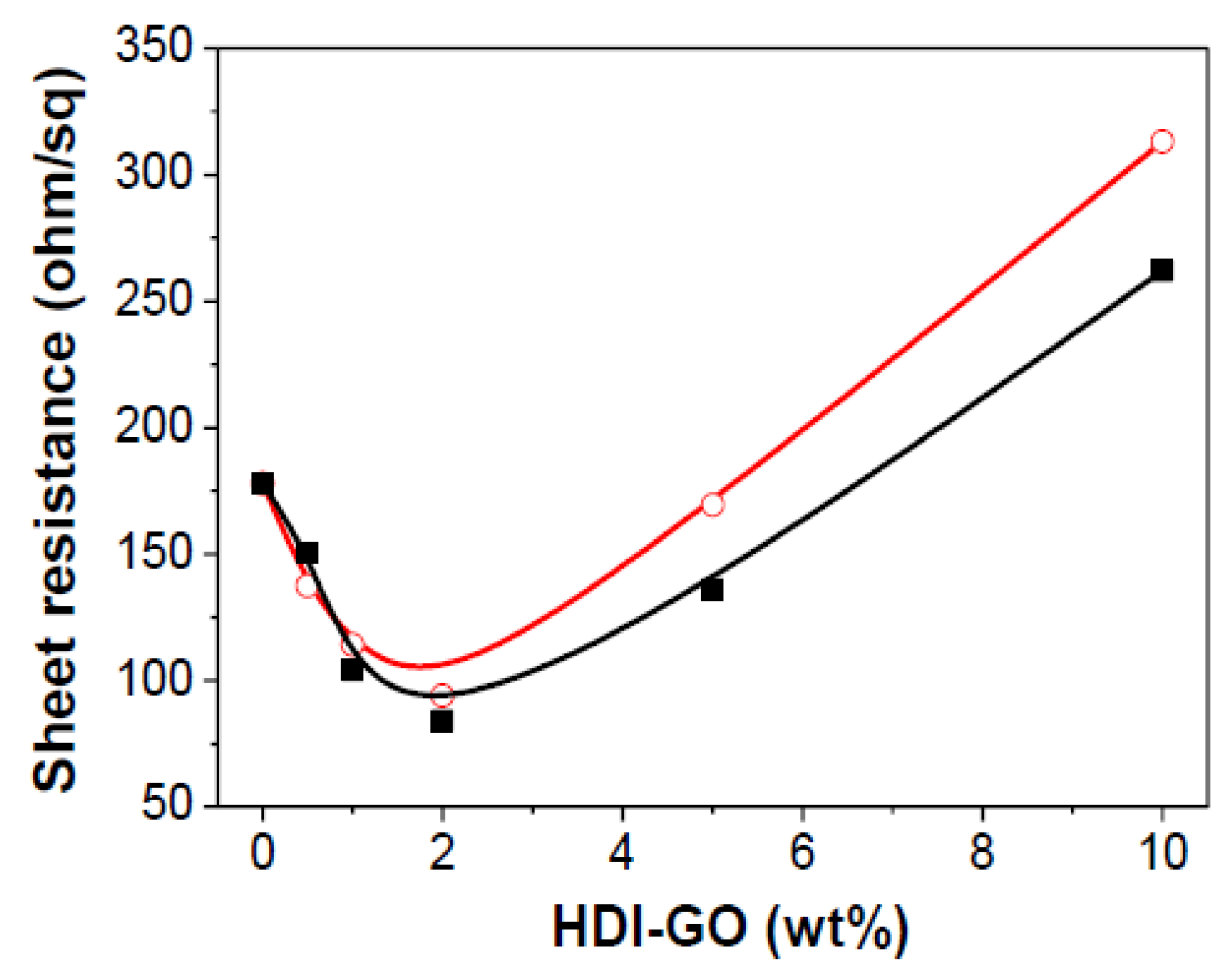
Sheet resistance of PEDOT:PSS nanocomposites reinforced with HDI-GO 6 (**open circles**) and HDI-GO 1 (**solid squares**) as a function of the HDI-GO content.

To investigate the effect of HDI-GO on the electrical properties of PEDOT:PSS, the sheet resistance (*R*_s_) data were measured, and the values obtained for nanocomposites reinforced with different amounts of HDI-GO 1 and HDI-GO 6 are compared in Figure 6. The *R*_s_ of raw GO and the HDI-functionalized GO samples was out of scale, since the functional groups on the GO surface disrupt the conjugated π-electron system of the graphene sheets, thus increasing the sheet resistance of pristine graphene, which is in the range of 18–300 Ω/sq depending on the synthesis method, number of layers, defect content, etc. [28]. 

On the other hand, the neat PEDOT:PSS film shows an *R*_s_ value close to 180 Ω/sq., which decreases gradually with increasing HDI-GO loading up to 2 wt %, leading to a minimum value of 84 Ω/sq for the nanocomposite reinforced with such an amount of HDI-GO 1. However, at higher nanofiller concentrations, *R*_s_ starts to increase, resulting in 1.7-fold for the HDI-GO 6 nanocomposite with the highest loading compared to the neat polymer. A comparable rise in *R*_s_ was also found for the reference P/GO (10 wt %) nanocomposite. Taking into account that HDI-GO samples display higher R_s_ than neat PEDOT:PSS, the reduction in sheet resistance found at low HDI-GO contents is an unexpected behaviour and could be explained considering the different factors that influence the charge hopping conduction mechanism in this conductive polymer, including grain size, doping and screening effects as well as conformational changes of the polymeric chains [43]. PEDOT and PSS are held by electrostatic attractions between the positive charges of PEDOT and the negatively charged sulfonyl groups of PSS, and display a coiled or core–shell structure, owing to the repulsion between long PSS chains. In the presence of HDI-GO, these ionic interactions are probably screened via formation of hydrogen bonds between its surface OH groups and the sulfonyl moieties of PSS (see Scheme 2). This would result in larger phase separation between PEDOT and PSS, linearly arranged PEDOT chains as well as more aggregated PEDOT chains on the nanocomposite surface [44]. The rearrangement of the PEDOT segments from coiled to linear or extended-coil structure would facilitate the inter-chain contact, thus promoting the charge hopping, and, consequently, the sheet resistance is reduced. Analogous behaviour of electrical conductivity improvement due to screening effects has been reported in the presence of polar solvents, like methanol [45]. Therefore, two opposite factors likely govern the electrical behaviour of the HDI-GO reinforced nanocomposites: The abovementioned conformational change of the PEDOT chains that favors charge conduction, and the presence of a nanomaterial with a higher sheet resistance than the matrix, which hampers the inter-chain charge movement. According to the experimental results, at low HDI-GO concentrations, the first factor seems to prevail, whilst at loadings higher than 5 wt %, the second one would predominate, thereby leading to an increase in R_s_. 

For the same nanofiller content, R_s_ is generally higher for nanocomposites filled with HDI-GO 6 compared to those with HDI-GO 1. Despite this, HDI-GO 6, with a higher FD, is more homogeneously dispersed within the matrix, and has less residual surface OH groups capable of interactions with the sulfonyl groups of PSS, hence the screening effect will be weaker, therefore leading to a higher sheet resistance. Further, HDI-GO 6 should have poorer electrical conductivity than HDI-GO 1, since the HDI treatment partly perturbs the aromatic π- system of the GO nanosheets.

It is important to note that the nanocomposites with low HDI-GO content display R_s_ values close to those reported for ITO films coated onto plastic substrates, such as polyethylene terephthalate (PET) [46], hence, they are well suited to use as transparent conductive electrodes in conventional panel displays, solar cells, touch panels, and so forth. Further, R_s_ values obtained herein for HDI-GO concentrations in the range of 0.5–5 wt % are lower than those reported for solution processed PEDTO:PSS/rGO films (2 × 10^2^–2 × 10^3^ Ω/sq) [47,48] or PEDOT:PSS/CNT films (≥500 Ω/sq) [40], which showed higher sheet resistance than the neat polymer, attributed to the poor nanocomposite quality due to non-homogenous dispersion of the CNTs or the r-GO sheets within the conducting PEDOT:PSS matrix. 

### 3.7. Mechanical Properties

**Figure 7:**
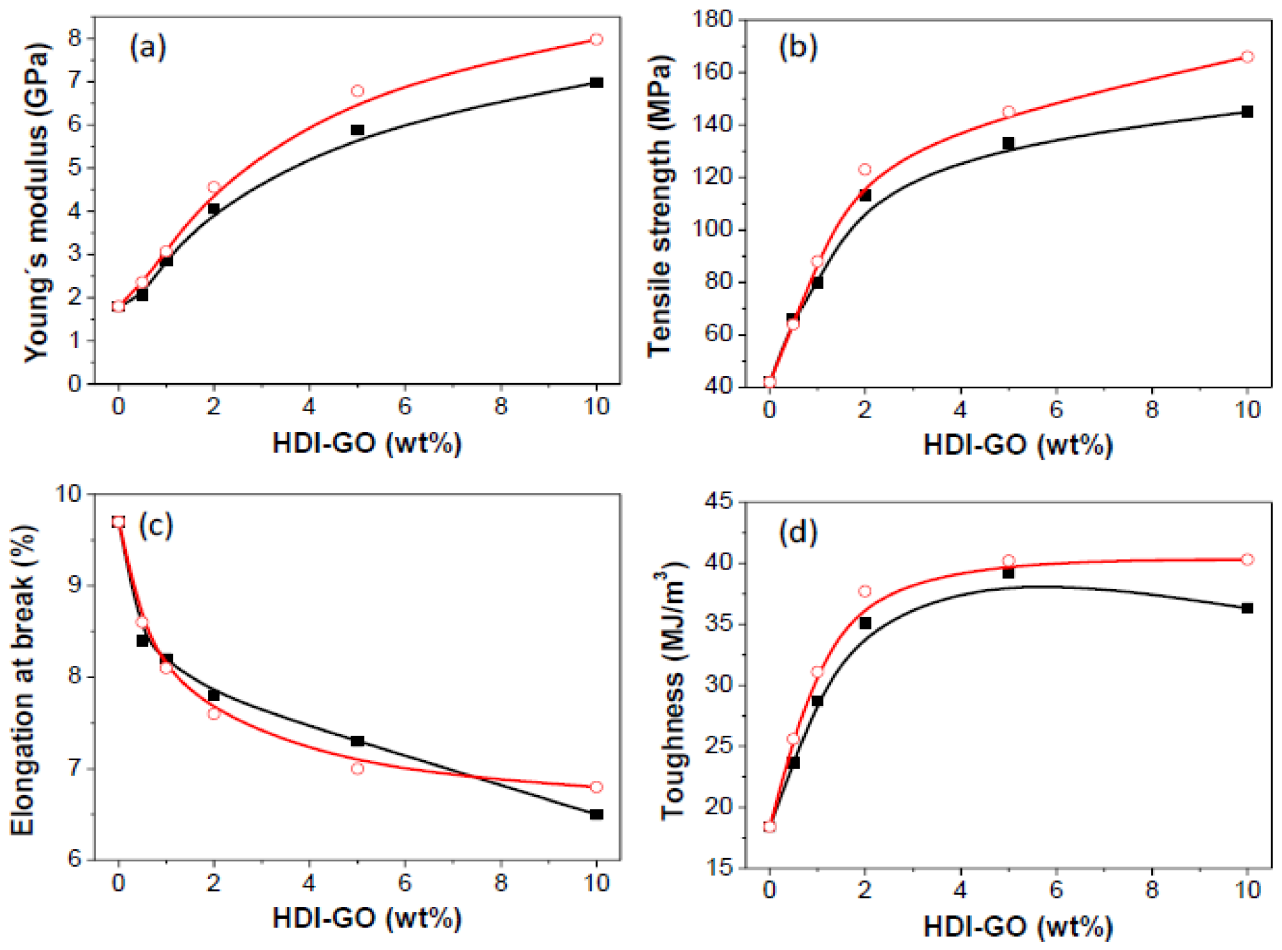
Mechanical properties of PEDOT:PSS nanocomposites reinforced with HDI-GO 6 (**open circles**) and HDI-GO 1 (**solid squares**) as a function of the HDI-GO content. (**a**) Young’s modulus; (**b**) tensile strength; (**c**) elongation at break; (**d**) toughness.

The mechanical properties of PEDOT:PSS/HDI-GO nanocomposites were investigated via tensile experiments, and their Young’s modulus, tensile strength, elongation at break, and toughness measured as the area under the tensile curve are compared in Figure 7. Neat PEDOT:PSS exhibits Young´s modulus and tensile strength values of 1.8 GPa and 42 MPa, respectively, consistent with the results reported previously [49]. The addition of HDI-GO 6 leads to strong improvements in both modulus and strength (Figure 7a,b), by up to 330 and 305%, respectively, at the highest loading tested. An analogous trend, albeit with slightly lower increases, is found for the nanocomposites reinforced with HDI-GO 1. For both types of nanocomposites, the rise is almost linear up to 2 wt % loading, while it is less pronounced at higher contents, and seems to level off at concentrations higher than 10 wt %. At low weight fractions, the effective reinforcing surface area of the individual and very well exfoliated GO nanosheets (Figure 1d) would be larger compared to the more stacked layers found at higher loadings, thus leading to a more pronounced reinforcement effect. The extraordinary modulus and strength improvements observed in these nanocomposites demonstrates the high reinforcing efficiency of HDI-GO, in particular that with the highest FD, likely arising from the combination of a random and very homogenous nanomaterial dispersion within the matrix (Figure 1e) and a very strong PEDOT:PSS-HDI-GO interfacial adhesion attained via hydrogen bonding, electrostatic, hydrophobic, and π-π interactions, as discussed earlier (Scheme 2), together with the high modulus of GO (~207 GPa [50]). Regarding the reference P/GO (10 wt %) nanocomposite, the improvements in modulus and strength were considerably smaller, around 185 and 120%, respectively, likely due to the presence of aggregates (Figure 1c) that reduce the GO-PEDOT:PSS interfacial area and limit the load transfer efficiency, combined with the weaker GO-matrix interactions; hence, poorer impregnation of the nanosheets by the polymer chains. 

Interestingly, the reinforcement effect found herein is stronger than that reported upon addition of comparable amounts of GO to other polymeric matrices, including poly(vinyl alcohol), polystyrene, poly(vinyl chloride), and poly(methyl methacrylate) [51]. Further, up to 2 wt % loading, the reinforcing efficiency attained with HDI-GO 6 is comparable to that observed for PEDOT:PSS nanocomposites filled with polyethylene glycol (PEG)-modified single-walled CNTs, despite the higher modulus of the CNTs (about 1 TPa) [52] compared to GO. All these facts corroborate the effectiveness of the HDI treatment and the solution casting process developed herein to improve the mechanical performance of PEDOT:PSS. 

Regarding the elongation at the break (Figure 7c), PEDOT:PSS presents a value close to 10%, which gradually decreases with increasing HDI-GO, the drop being ~29% for the composite with the highest HDI-GO 6 loading. This is the typical behaviour observed in nanofiller-reinforced polymer nanocomposites [53], since the fillers restrict the ductile flow of the polymer segments. Further, the strong PEDOT:PSS-HDI-GO 6 interfacial adhesion attained via hydrogen bonding and electrostatic, hydrophobic and π-π interactions contributes to the reduced plasticity. In general, the drop in ductility is smaller for the nanocomposites with HDI-GO 1 compared to those filled with HDI-GO 6, likely due to the weaker interactions with the matrix chains. In contrast, the reference P/GO (10 wt %) nanocomposite shows a more drastic diminution in the elongation at break value, about 64% compared to the neat polymer, since the aggregated GO nanosheets can intensely obstruct the plastic deformation of the matrix chains. Even so, the reductions in ductility found upon addition of HDI-GO are smaller than those reported for nanocomposites with similar amounts of PEG-modified CNTs embedded in PEDOT:PSS [52], likely due to the more homogeneous dispersion of the HDI-GO, since the presence of small CNT agglomerates strongly hampers the deformation of PEDOT:PSS. The combination of high strength and good ductility found for the nanocomposites developed in this work is interesting for applications in flexible electronics.

Another indication of an effectively reinforced nanocomposite is the toughness (the total energy required to break the composite estimated by the area under the stress-strain curve, Figure 7d), which is dependent on both the strength and the elongation at the break of the material. The change in toughness of the nanocomposite with the HDI-GO loading followed a similar trend to that described previously for the modulus and strength, even though the elongation at break decreased with increasing nanofiller concentration. The highest toughness was attained at 5 wt % HDI-GO 6 content, and is 122% higher than that of neat PEDOT:PSS (18.1 MJ/m^3^). Beyond this loading, the toughness remained almost constant for HDI-GO 6 nanocomposites or decreased slightly for those reinforced with HDI-GO 1. The trend found herein contrasts with that generally reported for GO-reinforced polymers [51], in which the toughness drops strongly at high nanofiller contents. The improved behaviour attained in this work is ascribed to a very homogeneous HDI-GO dispersion that minimizes the stress concentration nuclei, as well as an enhanced PEDOT:PSS-HDI-GO interfacial adhesion, that should provide an effective barrier for the propagation of cracks. This improvement is very significant from an application viewpoint. Conversely, the toughness strongly dropped for the reference P/GO (10 wt %) nanocomposite, by about 35%, since the aggregated GO nanosheets can act as stress-concentration points or crack initiators under applied loads, which adversely affects the ductility and toughness.

## 4. Conclusions

PEDOT:PSS-based nanocomposites reinforced with different amounts of hexamethylene diisocyanate (HDI)-modified GO have been prepared by a straightforward solution casting method, and have been characterized by means of a variety of techniques to investigate the effect of the HDI-GO concentration and the HDI-GO functionalization degree on the nanocomposite properties. According to SEM analysis, HDI-GO is better dispersed within the matrix than pristine GO, the dispersion being more homogenous upon increasing its functionalization degree. HDI-GO can strongly interact with PEDOT:PSS via hydrogen bonding, electrostatic, hydrophobic, and π-π interactions, as corroborated by the Raman spectra and XRD diffraction patterns. TGA results demonstrate that the addition of HDI-GO with a high FD significantly improves the thermal stability of PEDOT:PSS. Conversely, the optical transmittance slightly decreases with increasing HDI-GO concentration. The sheet resistance depends strongly on the amount of HDI-GO, showing a minimum value at 2 wt % loading. The nanocomposites incorporating the HDI-GO with the highest FD show a good combination of stiffness, strength, ductility, and toughness, and the best mechanical performance is found at 5 wt % loading. A compromise in the amount of HDI-GO nanosheets in the PEDOT:PSS matrix would be necessary to obtain optimal performance in terms of thermal, optical, electrical, and mechanical properties, with a view to use these nanocomposites in a variety of fields, in particular, in organic photovoltaics (OPV), organic light-emitting diodes (OLED), touch screens, panel displays, biosensors, and so forth. 

## Supplementary Materials

Supplementary materials are available online at http://www.mdpi.com/2073-4360/10/10/1169/s1.
