# Supplementary material for: High-Performance PEDOT:PSS/Hexamethylene Diisocyanate-Functionalized Graphene Oxide Nanocomposites: Preparation and Properties
Source: Polymers (Basel). 2018 Oct 20;10(10):1169. doi: 10.3390/polym10101169 (PMC6403542; doi:10.3390/polym10101169)
Supplement: Supplementary file 1 [file polymers-10-01169-s001.pdf]

Supplementary Materials

High-performance PEDOT:PSS/hexamethylene diisocyanate -functionalized graphene oxide nanocomposites: preparation and properties

José A. Luceño Sánchez[1], Rafael Peña Capilla[2], and Ana M. Díez-Pascual[1]*

[1] Department of Analytical Chemistry, Physical Chemistry and Chemical Engineering, Faculty of Biology, Environmental Sciences and Chemistry, Alcalá University, 28871 Madrid, Spain; jose.luceno@uah.es

[2] Department of Signal Theory and Communication. Polytechnic High School, Alcalá University, 28871 Madrid, Spain; rafa.pena@uah.es

*Correspondence: am.diez@uah.es; Tel.: +34-918-856-430

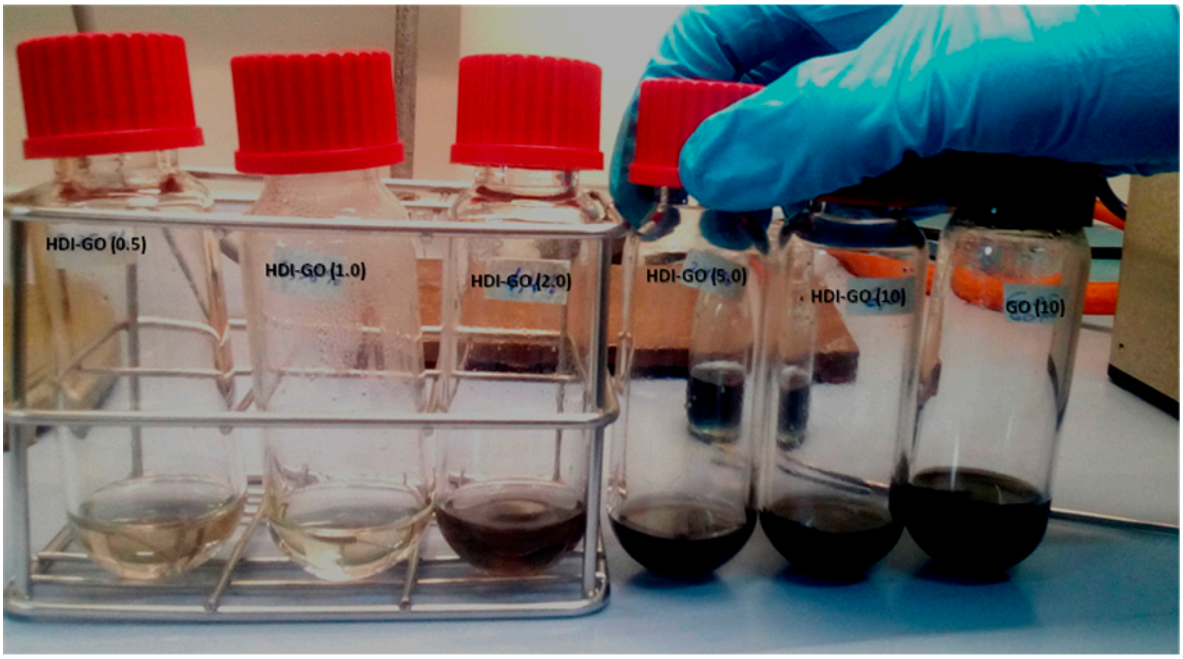

Figure S1. Photographs of the HDI-GO/PEDOT:PSS dispersions with HDI-GO 6 weight ratios of 0.5, 1.0, 2.0, 5.0 and 10 wt% and the reference sample with 10 wt% GO.

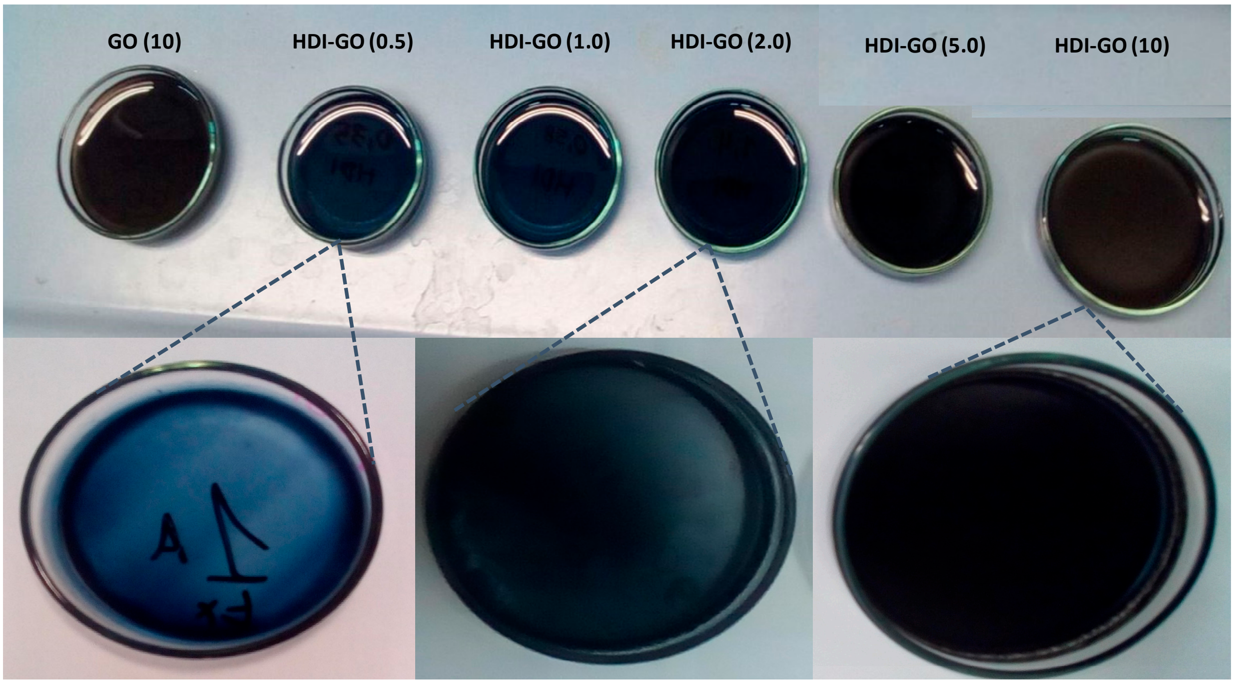

Figure S2. Photographs of PEDOT:PSS nanocomposites incorporating different GO or HDI-GO 6 contents obtained via solution casting.

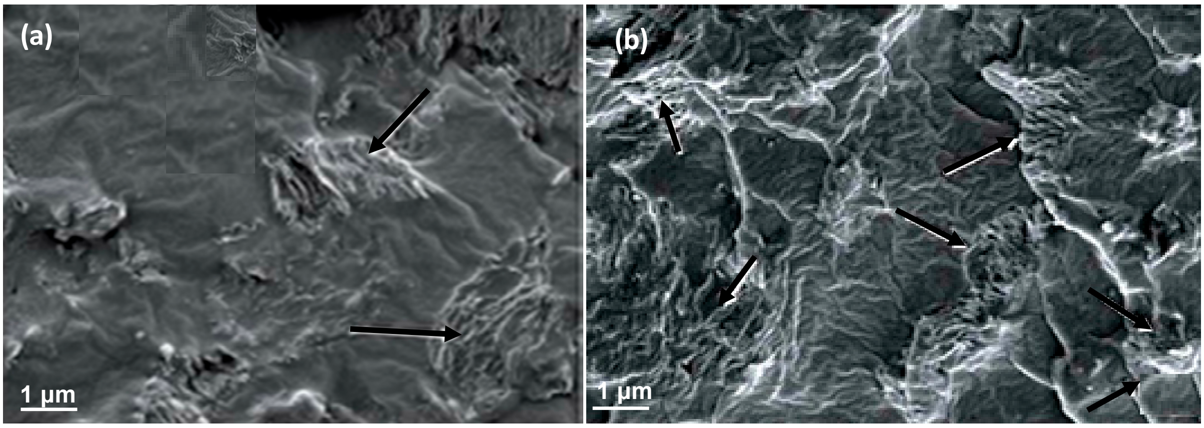

Figure S3. SEM micrographs of PEDOT:PPS/HDI-GO 1 nanocomposites with 0.5 wt% (a) and 10 wt% (b) nanomaterial loading. The arrows show HDI-GO nanosheets embedded within PEDOT:PSS.

Table S1. Data obtained from the Raman spectra and XRD patterns of PEDOT:PSS/HDI-GO nanocomposites.

Sample	$\nu_{c\text{-}c}$ (cm^{-1})	$\nu_{s\ c=c}$ (cm^{-1})	$\nu_{as\ c=c}$ (cm^{-1})	(020) (2θ)[a]	d_{002} (nm)
PEDOT:PSS	1356	1427	1550	25.95	–
P/GO (10 wt%)	1357	1427	1553	25.79	0.776
P/HDI-GO 1 (0.5 wt%)	1357	1428	1552	25.81	0.967
P/HDI-GO 1 (1.0 wt%)	1358	1429	1553	25.56	0.993
P/HDI-GO 1 (2.0 wt%)	1357	1428	1555	25.43	1.045
P/HDI-GO 1 (5.0 wt%)	1360	1430	1559	25.08	1.072
P/HDI-GO 1 (10 wt%)	1360	1431	1563	25.13	1.108
P/HDI-GO 6 (0.5 wt%)	1358	1430	1554	25.76	1.005
P/HDI-GO 6 (1.0 wt%)	1358	1433	1557	25.33	1.044
P/HDI-GO 6 (2.0 wt%)	1359	1434	1561	25.09	1.082
P/HDI-GO 6 (5.0 wt%)	1362	1435	1567	24.87	1.143
P/HDI-GO 6 (10 wt%)	1364	1435	1567	24.92	1.165

$\nu_{c\text{-}c}$: single C–C stretching; $\nu_{s\ c=c}$: C=C symmetrical stretching; $\nu_{as\ c=c}$: C=C antisymmetric stretching.
[a]position of the (020) plane of PEDOT:PSS; [b]interlayer *d* spacing values of (002) peak of GO.

Table S2. TGA data of PEDOT:PSS/HDI-GO nanocomposites.

**Sample**	**T_i (ºC)**	**T_{10} (ºC)**	**$T_{max(I,II)}$ (ºC)**	**R (wt%)**
PEDOT:PSS	130	227	249, 479	6.8
P/GO (10 wt%)	138	202	227, 448	5.2
P/HDI-GO 1 (0.5 wt%)	139	238	260, 496	7,1
P/HDI-GO 1 (1.0 wt%)	148	247	269, 513	7.9
P/HDI-GO 1 (2.0 wt%)	159	258	278, 534	8.5
P/HDI-GO 1 (5.0 wt%)	168	265	290, 549	9.8
P/HDI-GO 1 (10 wt%)	167	263	287, 548	10.2
P/HDI-GO 6 (0.5 wt%)	141	239	261, 498	7.3
P/HDI-GO 6 (1.0 wt%)	153	250	273, 517	7.6
P/HDI-GO 6 (2.0 wt%)	167	264	287, 540	8.7
P/HDI-GO 6 (5.0 wt%)	185	280	305, 563	9.9
P/HDI-GO 6 (10 wt%)	184	278	302, 560	10.5

T_i: initial degradation temperature at 2% weight loss; T_{10}: temperature of 10% of weight loss. T_{max}: temperature of maximum rate of weight loss. The subscripts I and II refer to the first and second degradation stages, respectively. R: residue at 700 ºC.
